# Efficacy and safety of open lung ventilation in patients with impaired peripheral chemoreflex sensitivity

**DOI:** 10.1186/cc14326

**Published:** 2015-03-16

**Authors:** N Trembach, I Zabolotskikh

**Affiliations:** 1 Kuban State Medical University, Krasnodar, Russia

## Introduction

Mechanical ventilation during anesthesia leads to the development of atelectasis, poor oxygenation and postoperative pulmonary complications. Application of PEEP and recruitment maneuver (RM) can significantly reduce the severity of atelectasis and improve lung function. But the application of this strategy often leads to hemodynamic instability, which may be associated with impaired reactivity of the cardiovascular system. The purpose of this study was to evaluate the efficacy and safety of RM in patients with increased sensitivity of peripheral chemoreceptors (SPCR), which reflects the decreasing reactivity of the cardiovascular system.

## Methods

We conducted a prospective study in 116 patients with high SPCR, evaluated using the breath-holding test. The test was performed by measuring of voluntary breath-holding duration (BHD) after two-thirds of maximal inspiration. The end of breath-hold was determined by a palpation of contraction of the diaphragm. BHD <38 seconds was the marker of high SPCR [1]. All patients received a major abdominal surgery and were randomized into an open lung ventilation group or a PEEP group. The concept of open lung ventilation was performed as follows: PEEP was increased from 4 to 10 cmH_2_O for three breaths, from 10 to 15 cmH_2_O for three breaths, and from 15 to 20 cmH_2_O for 10 breaths [2]. Then PEEP was reduced to 12 cmH_2_O. This RM was repeated every hour. In the PEEP group PEEP was maintained at 12 cmH_2_O during the whole anesthesia. Hemodynamics, blood gases and dynamic compliance were evaluated.

## Results

RM improved oxygenation compared with the PEEP group. The mean increase in the oxygenation index at the end of surgery was 31% (from 340 to 445 mmHg, *P *< 0.05), in the PEEP group the increase was less significant and amounted to 12% (from 330 to 370 mmHg, *P *< 0.05). Dynamic compliance increased by 35% in the RM group and did not change in the PEEP group. Hemodynamic changes at RM were more pronounced. So CI on average decreased by 34% (from 3.7 to 2.5 l/minute/m^2^) compared with 10% with no RM (*P *< 0.05), and SVR decreased by 19% (from 1,310 to 1,150 dyn × sec^-1^ × cm^-5^, *P *< 0.05), while in the PEEP group it did not change. No significant differences between groups in the incidence of complications, length of stay in the ICU and in the hospital were noted.

## Conclusion

RM patients with high SPCR and with reduced reactivity of the cardiovascular system improve lung function, but this is associated with the risk of hemodynamic instability.
